# Nano-Structured Lignin as Green Antioxidant and UV Shielding Ingredient for Sunscreen Applications

**DOI:** 10.3390/antiox10020274

**Published:** 2021-02-10

**Authors:** Davide Piccinino, Eliana Capecchi, Elisabetta Tomaino, Sofia Gabellone, Valeria Gigli, Daniele Avitabile, Raffaele Saladino

**Affiliations:** 1 Department of Ecology and Biology, University of Tuscia, San Camillo De Lellis, 01100 Viterbo, Italy; e.capecchi@unitus.it (E.C.); e.tomaino@unitus.it (E.T.); sofia.gabellone@studenti.unitus.it (S.G.); valeria.gigli@studenti.unitus.it (V.G.); 2 IDI Farmaceutici, Via dei Castelli Romani 73/75, 00071 Pomezia, Italy; davitabile@idifarmaceutici.it

**Keywords:** lignin nanoparticles, cosmetic and cosmeceutical applications, antioxidant activity, UV shielding activity, sunscreen formulations

## Abstract

Green, biocompatible, and biodegradable antioxidants represent a milestone in cosmetic and cosmeceutical applications. Lignin is the most abundant polyphenol in nature, recovered as a low-cost waste from the pulp and paper industry and biorefinery. This polymer is characterized by beneficial physical and chemical properties which are improved at the nanoscale level due to the emergence of antioxidant and UV shielding activities. Here we review the use of lignin nanoparticles in cosmetic and cosmeceutical applications, focusing on sunscreen and antiaging formulations. Advances in the technology for the preparation of lignin nanoparticles are described highlighting structure activity relationships.

## 1. Introduction

### 1.1. Drawbacks of Current Antioxidant and UV Shielding Ingredients in Sunscreen Formulations

The harmful consequences of prolonged exposure of human skin to sunlight have long been explored [1]. UV radiation (190–400 nm) is the main cause of generation of radical reactive oxygen and nitrogen-centered species and related oxidative stress. Radicals are responsible for lipid peroxidation and degradation of elastin and collagen, promoting both loss of skin elasticity and aging [2]. In addition, they react with DNA promoting apoptosis, genetic mutation, and cancer [3]. In this latter case, the radical damage is related to melanoma, the most aggressive form of skin cancer, arising from melanocytes resistant to chemotherapy [4,5]. Sunscreen formulations are designed to protect the skin from solar radiation and possibly from melanoma. They contain active ingredients capable of absorbing (organic chemical filters, OCFs) or reflecting (physical mineral filters, PMFs) the UV radiation. PMFs are generally used as nanoparticles since the nanoscale improves the reflectance activity, as well as the consumer cosmetic acceptance [6]. In a typical high sun protection factor (SPF) sunscreen, the final concentration of PMFs and OCFs can reach 45% by weight [7]. In addition, UV boosters are used to further increase the SPF by physical effect, tuning the ratio between the UV shielding efficiency and the total UV filter concentration [8]. UV boosters are usually composed of plastic micro-sized spheres from petroleum origin, such as styrene/acrylate copolymers, that are well recognized as a waste in water pollution [9].

High amounts of PMFs, OCFs, and UV boosters, are detected in marine environments [6] as a consequence of their release from cosmetics, producing submicronic aggregates in freshwater, or alternatively, sediment settles in seawater [10]. Consequently, the growing consumption of sunscreens has gradually led to a significant increase in coastal pollution with a deleterious impact on marine organisms [11]. 3-Benzylidene-camphor (3-BC) and 3-(4-methyl benzylidene)camphor (4-MBC), which belong in the OCFs family, induce severe and fast coral bleaching, altering the symbiosis between coral and zooxanthellae, and inhibiting the reproduction of both oligochaete *Lumbriculus variegatus* [9] and marine phytoplankton [12]. Moreover, they are concentrated in tissues of aquatic organisms (mussels, crustaceans, eels, fishes, marine mammals, and pelagic birds) due to the high lipophilicity [13]. In a similar way, PMFs are harmful to marine ecosystems. Zinc oxide nanoparticles contributed significantly to the whitening of the *Acropora* spp. [14], and titanium oxides negatively affect dinoflagellates, fish, algae, and marine invertebrates [15].

**Figure 1:**
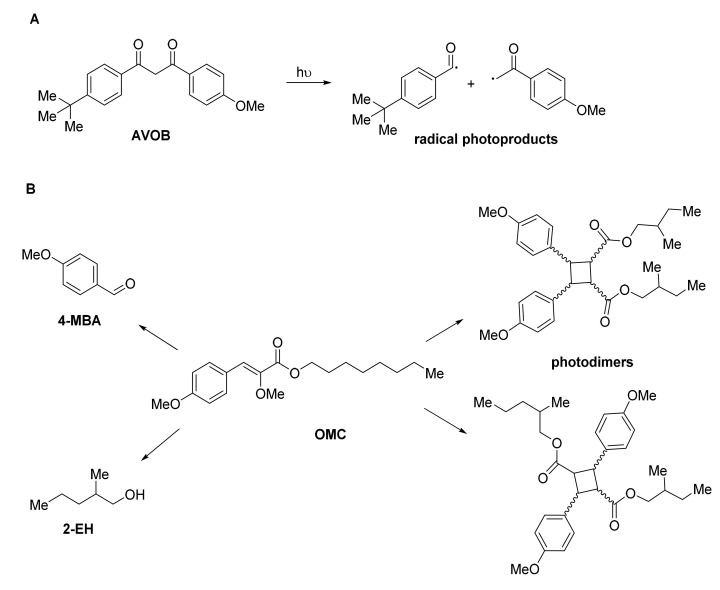
Schematic representation of photodegradation processes operative in the case of avobenzone (AVOB) (**A**) and octyl methoxycinnamate (OCM) (**B**).

Risks for human health are also associated to UV filters [16] as a consequence of phototoxic and photoallergic responses and, in the long term, photoaging and cell damage [17]. The absorbance spectrum of OCFs changes during the UV exposure time as a consequence of structural transformation and degradation processes [18]. These processes can produce free radicals and interaction with other sunscreen ingredients and skin constituents (e.g., lipids, proteins, and nucleic acids), altering the absorbing property of the sunscreen and inducing oxidative damage [19]. Avobenzone (AVOB), that is a widely recognized OCF in commercial sunscreens, demonstrated photo instability after prolonged UV exposure to yield radical photoproducts (Figure 1A) [20], and octyl methoxycinnamate (OMC), the most used UV-B filter, is photo-unstable after exposure to sunlight, undergoing photodimerization and loss of shielding efficiency. In this latter case, the photoproducts 4-methoxy benzaldehyde (4-MBA) and 2-ethylhexanol (2-EH) are also produced (Figure 1B) [21,22]. Moreover, benzophenone-3 (BP-3), and octocrylene (OC) are able to penetrate in the epidermal nucleated layers generating in situ radical species after UV irradiation [23]. 

Despite photodegradation and generation of radicals, OCFs cause other drawbacks for human health. For example, AVOB promotes obesogenic phenotypes in normal human epidermal keratinocytes (NHEKs), increasing the gene transcription of peroxisome proliferator-activated receptor γ (PPARγ) and fatty acid-binding protein [24], and affecting diabetes [25] with disruptive endocrine effects [26]. The toxic effect of some of the most representative OCFs are summarized in Table 1.

### 1.2. Alternative UV Shielding and Antioxidant Ingredients in Sunscreen Formulations

The use of PMFs and OCFs is regulated by directives from different agencies, such as the Food and Drug Administration (FDA) and the European Chemicals Agency, in order to adequate the UV protection to minimal side effects for health [33]. The use of alternative eco-friendly and natural ingredients, deprived of toxic effects, is strongly recommended and foreseen in future formulations. 

The toxicity and pollution effects of actual commercial UV filters increased the interest for eco-friendly and natural alternatives [34]. Their use is considered an advantage not only for the ability to improve the SPF value in safer formulations, but also in improving the photostability of traditional UV filters [35]. Secondary metabolites of the cell, such as terpenoids and products of the shikimic acid and polyketide pathways, can play a relevant role bearing conjugated double bonds and aromatic pharmacophores with high UV absorbing and antioxidant properties [36]. They have been selected during molecular evolution by plants and microorganisms in developing defense strategies to minimize the damage of UV radiation and chemical oxidative stresses [37,38,39]. Sunscreen formulations containing mixtures of secondary metabolites from plants, algae, and fungi, as well as from cyanobacteria [40], showed a higher shielding effect with respect to commercial filters. In addition, mycosporins and mycosporin-like amino acids (MAAs) [41] produced by marine phototrophs (dinoflagellates, cyanobacteria, and macro algae) [42] are able to convert radiative energy in thermal energy without generation of radical species [43]. Sunscreen formulations containing MAAs from *Porphyra umbilicals* showed a high protective effect on fibroblasts and keratinocytes exposed to UV-A [44]. Mixtures of secondary metabolites from the spent fraction of coffee grounds and green coffee oil (GCO) have a synergistic effect with traditional OCFs and increase the SPF value of sunscreen formulations (+20%) [45]. The protective effect from UV-B exposure by grapes wine extract of Jacquez (namely JW-E), containing high level of proanthocyanidins, anthocyanins, and hydroxycinnamic acids, has been evaluated by three-dimensional tissue cell model of the human epidermis [46]. Finally, the water resistance of OCFs is improved by natural waxes that stabilize the SPF value of sunscreen formulations after immersion in water [47]. Waxes also increase the SPF value and improved the photostability of OCFs and PMFs after UV irradiation [35]. Despite exhibiting strong sun protection effect, most crude plant extracts are insoluble in water, thus hindering their practical applications.

## 2. Lignin as a Novel Eco-Friendly Sunscreen Ingredient

The development of sunscreen formulations based on renewable and recyclable resources received a great interest, mainly due to circular economy and green chemistry concerns. In this context, lignin, the most abundant polyphenol in nature, is increasingly becoming one of the main protagonists since it is recovered in large amounts as a low-cost waste from pulp and paper industry and biorefinery [48]. The low environmental impact of lignin extraction and purification technologies has been reported and discussed, focusing on the expected benefits of lignin as a high added value material [49,50,51]. Current and potential application of lignin has been reviewed [52,53,54,55,56], focusing on power fuel and syngas production (Fischer–Tropsch synthetic fuels), material science and fine-chemicals, specialties, and commodities preparation [57,58]. Lignin confers rigidity and microbiological and mechanical resistance to lignocellulosic materials [59]. In addition, it shows low-medium UV-shielding [60], antioxidant activities [61,62] and biocompatibility [63]. The lack of toxicity of lignin has been reported by in vivo toxicity assessment in embryonic zebrafish (*Danio rerio*) [64]. The efficacy of lignin as an alternative sunscreen ingredient depends on its origin and composition, as well as from the structural order and dimensional scale [65]. The main lignin functions in sunscreen cosmetics are summarized in Table 2. The next paragraphs will report about these specific aspects. 

### 2.1. Structure, Availability, and Green Application of Lignin

**Figure 2:**
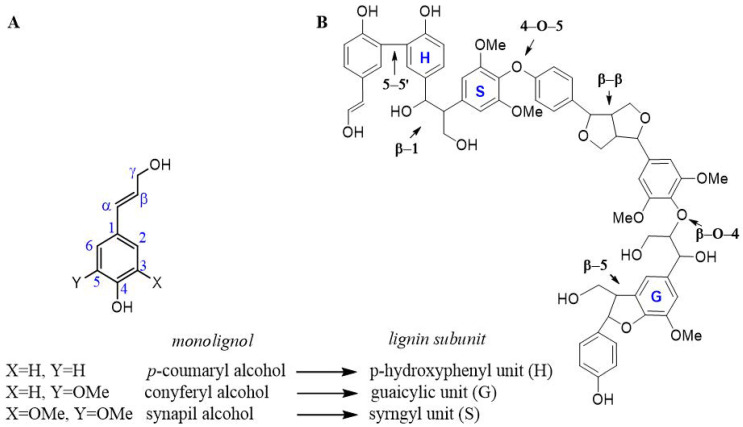
Chemical structure of the main components in lignin. (**A**) Monolignols; (**B**) representation of the main type of inter-units in lignin.

Lignin is an important source of phenolic compounds [71]. It is one of the main components in the plant cell wall beside cellulose, hemicellulose, pectin, and extractives. This polymer is biosynthesized by a cascade of oxidative radical couplings, involving three phenylpropanoid monomers (monolignols), namely *para*-hydroxyphenyl (H), syringyl (S), and guaicyl (G) alcohols (Figure 2A) [72]. These compounds differ in the degree of methoxylation of the aromatic ring and are present in the polymer in a different ratio depending on the plant species considered [59]. The biosynthesis of monolignols starts in the cytoplasm, from which they are successively transported to the apoplast and delivered in different zones of the secondary area (central lamella and secondary wall of the xylem) [73,74]. The physiological significance of this distribution is apparently simple: differential targeting allows for the construction of lignified cell walls with distinct biophysical properties [75].

The first step of the lignification process is the dehydrogenation of monolignols to corresponding phenoxy radicals plant cell-wall oxidases (lignin-peroxidase LiP, manganese-peroxidase MnP, hybrid LiP-MnP, versatile-peroxidase VP, and laccase) [72]. Monolignols form a network of inter-unit linkages to yield relatively low molecular weight oligomers involved in the formation of stable supramolecular π-π aggregates into a complex 3D molecular architecture [76]. The main types of inter-unit linkages are the alkyl-aryl ether β-O-4, phenylcoumaran (β-5), 1,2-diaryl-propane (β-1), diaryl (5-5), diaryl ether (4-O-5), pinoresinol (β-β), and dibenzodioxin structural residues (Figure 2B).

Lignin is classified into three basic types depending on the number and quality of sub-units: softwood, hardwood, and grass lignin (Table 3). In addition, some unconventional types of structural motif, such as caffeyl lignin units (C-lignin), were also found [77].

Different technologies are available to separate wood components, and they can be classified depending on the scale of the treatment [81]. At the analytical scale (from mg to gram), milled wood lignin (MWL) is considered the most reliable model of native lignin. MWL is produced from wood chips by extraction with 1,4-dioxane and successive precipitation in water [82]. At the industrial scale (ton scale), two main types of lignin are prevailing: sulfur-containing lignin and sulfur-free lignin. Sulfur-containing lignin include lignosulfonate (LS) and kraft lignin (KL), which are produced in a strong alkaline and oxidative medium. The second category includes soda lignin (SL), organosolv lignin (OL) ionic liquid lignin (ILL), and steam-exploded lignin (SEL) [83]. 

Sulfur-free lignins have been applied in the production of probiotics for animals due to their capacity to improve the growth of beneficial bacteria [84,85], influencing the absorption of bile acids in the lipid metabolism [86,87]. In addition, LS is used as a binder, dispersing agent, emulsifier, and heavy metal sequestrant in the design of advanced materials and wastewater treatment, as in the case of absorption of toxic chromium, copper, cadmium, lead, zinc, nickel, cobalt, and mercury ions [88]. From the biological point of view, KL and SEL showed antioxidant activity in preserving human red blood cells [89]. This activity was associated to non-etherified phenolic hydroxyl groups, ortho-methoxy groups, and aliphatic hydroxyl groups in the side chain. In particular, ortho substituents, such as methoxy groups, stabilize phenoxy radicals by resonance and hinder their propagation. Conjugated double bonds can also improve the antiaging activity by extensive electronic delocalization. In some cases, the antioxidant activity was associated to antiviral [90], antimicrobial, and anticancer properties [91].

### 2.2. The Technology for the Self-Assembling of Lignin Nanoparticles

The low solubility of lignin represents a limit for its application in the cosmetic and cosmeceutical formulations [92]. This bias can be solved by the self-assembly of the native polymer into highly ordered lignin nanoparticles (LNPs) [93]. LNPs are characterized by improved chemical and physical properties compared to the native state [94]. Their size tunability and spherical shape allowed to broaden the fields of application, encompassing drug delivery systems [95], flame retardant [96] and reinforcing materials [97], and waste treatment [98]. Remarkably, LNPs show improved UV shielding [94] and antioxidant properties [99] (see next paragraph). Table 4 reports the major technologies available for the production of LNPs, the relative experimental conditions, and some of their specific applications. Briefly, the following technologies have been reported for the preparation of LNPs: (i) solvent exchange precipitation method where the starting solvent was slowly replaced by an anti-solvent by dialysis; (ii) treatment of lignin solution by adding strong acid; (iii) aerosol flow reactor equipped with collision-type jet atomizer; (iv) microchannel reactor in which the lignin solution is continuously mixed with the antisolvent; (v) CO_2_ compressed fluid as antisolvent; (vi) flash-precipitation by fast adding of anti-solvent in lignin solution; and (vii) mini-emulsion between oil-phase and aqueous lignin solution under ultrasound treatment followed by polymerization with cross-linker agent. The formation of LNPs is influenced by different experimental parameters, including the physical properties of the medium (solvent, pH, temperature, stirring speed), and the chemical structure of the starting substrate (composition, molecular weight, polarity, and impurities). Among them, the pH value controls the ionization of the phenolic moieties, which in turn affect inter- and intra-molecular electrostatic interactions in the polymer. Recent studies based on light scattering analysis and pulse field gradient NMR, showed that the KL self-association process occurs below pH 9.0–11.5, the process being more efficient in water than in organic solvents [100]. In addition, a low value of temperature favors the aggregation [101], and a combined effect of temperature and pH can finely control the particle dimension [102]. As a general trend, softwood lignins show a higher degree of aggregation with respect to the hardwood counterpart, suggesting the greater presence of efficient intermolecular HOMO-LUMO interactions [103]. 

The structuration process of LNPs is driven mainly by noncovalent interactions, including H-bonding, van der Waals forces, and π-π stacking aggregation between the aromatic rings of different molecules [104]. Although no studies have been carried out on the specific role played by H-bonding in the self-assembly of lignin, this is commonly cited as a relevant contributing interaction due to its directional nature [105,106]. In accordance with this hypothesis, the formation of H-bonding network affects the mechanical properties of KL gels [107]. The specific role of the Van der Waals forces in tuning the macro-syneresis of KL gel has been also reported [108].

### 2.3. Antioxidant Activity

**Figure 3:**
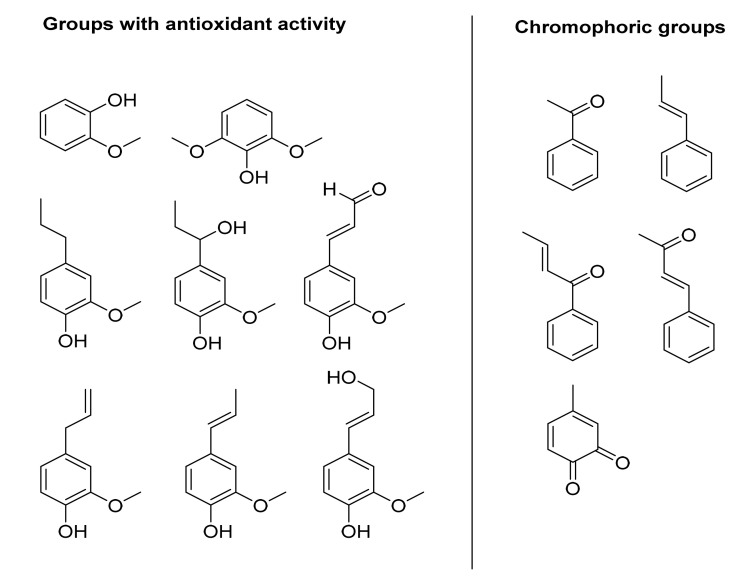
Classification and structural representation of lignin sub-units with antioxidant activity and chromophoric groups.

Radical species take part in various degenerative processes, including aging and inflammatory response triggered by UV exposure [123]. For this reason, antioxidants are common ingredients in sunscreen formulations [124]. Lignin shows antioxidant activity due to the presence of the phenolic pharmacophore able to scavenge reactive radical species with formation of highly stabilized mesomeric forms. Figure 3 describes the main phenolic sub-units responsible for the radical scavenger activity. Phenolic hydroxyl groups, ortho-methoxy groups, and aliphatic hydroxyl groups play a crucial role in this activity. In particular, ortho substituents, such as the methoxy groups, stabilize phenoxy radicals by both resonance and steric hindrance effects. 

The demethylation process of these groups, as well as the presence of conjugated double bonds, further increase the antioxidant activity by increasing the total amount of OH and favoring extensive electronic delocalization. Table 5 reports the antioxidant activity of a panel of low molecular weight compounds commonly considered as simplified models of lignin, as evaluated by the DPPH assay [103]. As a general trend, the antiradical power (ARP; defined as the reverse of the dose inhibiting the 50% of the DPPH radical) and the number of reduced DPPH units (NRD; which represents the moles of DPPH reduced per mole of compound) show that syringyl-like derivatives have higher antioxidant activity than guaiacyl and para-hydroxy phenyl counterparts (Table 5, entry I versus entry II). In addition, lower values of ARP and NRD were observed when a carbonyl moiety was present in the phenylpropanoid side-chain (Table 5, entry III, IV), while the presence of a conjugated double bond system increased the antioxidant activity (Table 5, entry VII, VIII, IX, and X) [125].

In addition to the capacity of sequestering radical species, lignin is promising to inhibit lipid peroxidation and, from a general point of view, the oxidative degradation of other cosmetic ingredients. For example, the ability of KL to inhibit the undesired radical production by PMFs (e.g., TiO_2_) in sunscreen formulations has been demonstrated [126]. Interestingly, the antioxidant activity of LNPs was different order of magnitude higher than that of native lignin [94,127]. This behavior can be explained by the higher density of phenolic and carboxylic groups on the surface of LNPs with respect to the native polymer [128], as well as by the occurrence of favorable electron transfer processes between the ordered π-π stacked aromatic moieties [117]. In order to further increase the antioxidant activity, LNPs with mixed adsorbed polyphenols were prepared, highlighting the synergistic effects among the components [129]. The application of LNPs as UV filter or, alternatively, UV booster in broad-spectrum sunscreen formulations has been explored [130,131,132], confirming the beneficial role of lignin when used in the nanoscale form.

### 2.4. UV Shielding Activity

**Figure 4:**
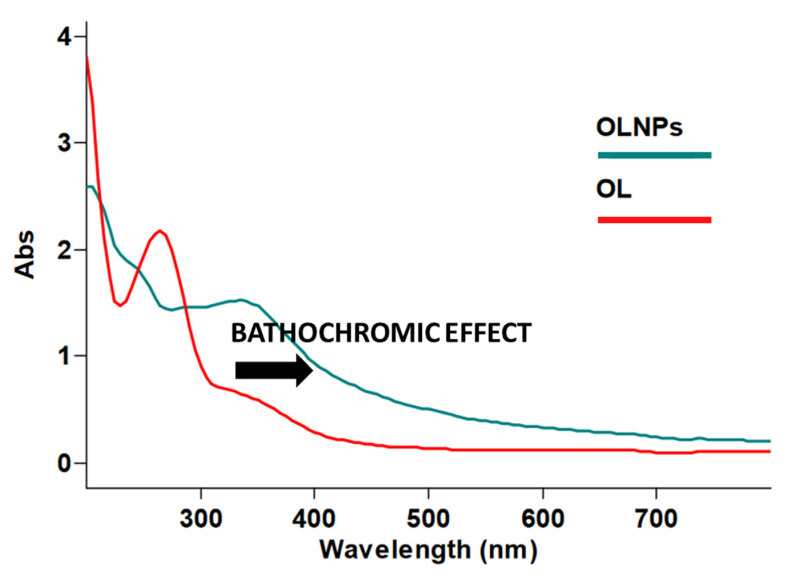
UV-VIS assay to evidence of bathochromic effect of organosolv lignin nanoparticles (OLNPs; green line) after the nanostructuration of starting technical organosolv lignin (OL; red line).

The Sun emits electromagnetic radiation in three ultraviolet (UV) wavelength areas. Rays with the shortest wavelength (UV-C, 100–290 nm) are captured by atmosphere, UV-A radiation at medium wavelength (UVA, 290–320 nm) and longer wavelength (UV-B, 320–400 nm) reach the surface of Earth. Excessive exposure to UV-B causes sunburn, while UV-A penetrates deeper into the skin. Both UV-A and UV-B are causing agents for DNA damage and cancer [133]. Lignin shows a broad absorption range in the UV region due to the presence of different chromophores and auxochromic groups (Figure 3), the maximum of absorbance being located at 283 nm [134,135]. Auxochromic groups (from ancient Greek αὐξάνω auxanō “increase” and χρῶμα chrōma “color”) are groups of atoms bearing non-bonding electrons (OH, OCH_3_, NH_2_, CO, SH, and SCH_3_) able to increase the chromophore effect. The contribution of auxochromic groups in lignin to generate a bathochromic effect (redshift phenomena) is reported [136] (Figure 4). LNPs are characterized by improved UV-shielding properties in relation to the native counterpart. For example, KL nanoparticles showed a higher absorption efficacy (up to 30%) than native KL, associated to the presence of a larger absorption band in the region of the longest wavelengths [94,137]. This effect is mainly due to the occurrence of π-π stacking interactions between the aromatic moieties, that can form two main types of aggregates, namely sandwich-type (H-orientation) and head-to-tail (J-orientation) aggregates. The J-orientation (water media) is the most efficient to decrease the energy gap for the π-π* electronic transition, thus enhancing the absorption efficacy of UV photons [138]. In addition, charge transfer complexes between electron-donating phenolic groups and electron acceptor ortho-quinones moieties can further increase the UV photo-absorbing capacity of LNPs [139].

Notably, experimental data highlighted that the UV-absorbing capacity of lignin increases during the exposition to radiation as a consequence of the formation of novel chromophores, such as quinonoid units [140]. The negative charged surface of LNPs can be further functionalized by consecutive deposition of natural macromolecules or polyelectrolytes with opposite charge by the layer-by-layer technique (LbL) [141,142]. In this way, mixed LNPs containing layers of other natural polyphenols, such as tannic acid, showed enhanced UV shielding properties, by occurrence of a synergistic effect between the aggregate polymers [116]. Finally, the cavity of LNPs can be exploited for the physical encapsulation of both OCFs and PCFs to yield functionalized LNPs with UV shielding properties higher than the parent compounds. One example of this strategy is represented by the encapsulation of TiO_2_ inside lignin-based colloidal nanoparticles to afford a stable UV filter deprived of undesired side-chain catalytic effect in the generation of radical species [143].

### 2.5. Other Physical and Chemical Properties of LNPs Useful in Sunscreen Formulation

LNPs are characterized by a large panel of physical and chemical properties useful for the design of sunscreen formulations, encompassing emulsion stabilizer, antimicrobial, and chelating properties. These properties will be explored in the following paragraphs.

#### 2.5.1. Emulsion Stabilizer Properties

The emulsions are unstable systems due to the high surface energy exerted between the two immiscible phases. For this reason, the presence of a surfactant able to reduce the surface energy is required [144]. Most surfactants derive from non-renewable precursors and are generally not biodegradable [145]. Lignin is considered an amphipathic polymer due to the presence of both hydrophilic and hydrophobic components. In addition, the presence of ionizable groups make it an efficient stabilizer by the occurrence of electrical repulsion effects [146]. A case of study for the application of LNPs as a stabilizer is represented by the production of Pickering emulsions, which were characterized by a higher deformation resistance compared to that stabilized with conventional surfactants. This effect was associated to the barrierless adsorption of the LNPs at the interfaces of two immiscible liquids, thanks to their partial wetting properties [147]. LNPs were also efficient Pickering emulsifiers to stabilize oil emulsions in water (O/W) in the formulation of sunscreen containing low soluble organic substances [148].

#### 2.5.2. Antimicrobial Properties

Lignin is characterized by antimicrobial activity as a consequence of the capacity to interact with the bacterial cell causing lysis with consequent release of the cell content. KL is effective against *Erwinia carotovora* and *Xanthomonas campestris pv. vesicatoria* (but not against *Pseudomonas syringae*), while AL showed antimicrobial activity against *Escherichia coli, Staphyloccocus aureus*, and *Pseudomonas* [149]. In particular, the C=C double bond and γ-methyl groups in the side chain confer to lignin a higher antimicrobial activity than phenolic and aliphatic groups [150]. Again, LNPs showed higher antimicrobial activity than the native counterpart, as a consequence of the highest contact surface area available for the interaction with the microorganisms [134], associated to the possibility to penetrate into the bacterial cell. In addition, LNPs favored the damage of the bacterial cell by electron transfer processes able to generate local radical species [115]. Examples of the synergy activity between silver nanoparticles [150] and lignin against Gram-positive and Gram-negative bacteria have been reported, focusing on the role of lignin as a recognizer and delivery system for the controlled release of the silver ion [151]. In this latter case, the amount of silver ions used in the treatment was 10 times lower than conventional materials, reducing the known negative impact of silver wastes on the environment. Examples of the use of lignin as a green antimicrobial ingredient in the formulation of cosmetics are reported, as in the case of the reduction or substitution of high environmental impact antimicrobial agents, such as phenoxyethanol, hydroxybenzoates, and triclosan [68].

#### 2.5.3. Chelating Properties

Chelating agents are ingredients able to complex metal ions in a stable way. These compounds play a crucial role in the stability and efficacy of cosmetics since the chelation mechanism stabilizes metal ions by preventing them from reacting with other substances and skin. The adsorbing capacity of lignin has been studied against different metal species, including chromium, copper, cadmium, lead, zinc, nickel, cobalt, and mercury [152,153]. The chelating process occurs by coordination of the metals with Lewis basic sites in lignin, such as carbonyl, carboxylic, and phenolic groups [154]. The adsorption was found to be pH dependent, being favored by deprotonation of the active groups in lignin [155]. In this context, the use of lignin to replace commercial chelating agent characterized by a well-known environmental pollution impact (e.g., EDTA) has been reported [156].

## 3. Color Agreeableness of Lignin

The dark color of lignin, that is mainly due to the harsh conditions of physical and chemical treatments (e.g., high temperature and oxidative transformations), hinders the promotion of lignin-based cosmetics on the market [157]. The principal methods to assess the lignin color are the Munsell and CIELAB procedures [158]. The Munsell color system determines the classification of the color by measuring the human perceptual response, and consists in the evaluation of three independent variables, represented by the color hue (measured in degrees on a horizontal circle), saturation (measured radially from the neutral gray axis outwards), and brightness (measured vertically on the gray axis from 0 for black and to 10 for white) parameters. This method is accurate and quantitative, but it is limited by a laborious visual matching. The CIELAB method is based on a spectrophotometer analysis in two different modes: (i) specular component included (SCI); and (ii) specular component excluded (SCE) modes. SCI is used to evaluate the actual color using both specular and diffuse reflected light, while SCE determines the color by excluding any specular reflected light. In the International Commission on Illumination L*a*b* (CIELAB) color space, the L* value represents a bright behavior of a sample as follows: white when L* = 100 and black when L* = 0; +a* is a red shade and −a* is a green shade; +b* is a yellow shade and −b* is a blue shade. A total color difference value (ΔE) is defined as following Equation (1):

(1) $$\Delta E=\frac{\left[\left(\Delta L*\right)2+\left(\Delta a*\right)2+\left(\Delta b*\right)2\right]}{2}$$

where ΔL*, Δa*, and Δb* are the differences in L*a*b* values between a reference and a sample. In order to enhance the color agreeableness of lignin, the chromophore and auxochrome groups should be blocked or cleared. This is of particular relevance in the case of sunscreen formulation. The main techniques applied to lighten lignin are described in the following paragraphs.

### 3.1. Drying of Lignin

Drying of lignin involves the control of the organization of the polymer structure at the morphological level by removal of the water molecules included in the sample [159]. An appropriate drying procedure can significantly reduce the dark color of lignin without extensive structural modification of chromophores and auxochrome moieties. This procedure has been applied in the preparation of light-colored sun creams, the control of the color intensity depending on the specific drying method (i.e., oven, vacuum, freeze, and spray drying) [160]. Oven and vacuum drying procedures favored the formation of large particles with a glossy surface and a dark color, while spray and freeze drying make the lignin as a lighten colored fine powder. In addition, a correlation between the particle size and the lignin color was observed, small-sized particles being less colored.

### 3.2. Fractionation of Lignin with Solvents

Light-colored lignin nanoparticles (CEL-NP) have been obtained from rice husks through initial extraction of cellulolytic enzyme lignin (CEL) followed by the solvent shifting procedure [161]. The color of CEL-NP and CEL was evaluated using the L* a* b* (CIELAB) color space method and compared with lignin from rice husks without cellulase treatment (RH) and organosolv lignin (OL), as references. The brightness (L*) and redness (a*) values of CEL were similar to those of RH as a consequence of the low effect of cellulase activity on phenolic and quinoid chromophore groups. The L* value of CEL was higher than that of OL and the a* value of CEL was lesser than OL. This is because CEL was prepared under much milder conditions than OL. As expected, the nanostructuration process further increased the lightness of the sample (CEL-NP). The effect of mixture of organic solvents in the lightness of lignin has been also reported [162].

### 3.3. Heat Treatment

The thermal processing of lignin involves the cleavage of β-O-4 linkages and demethoxylation. Usually, this process produces condensed structures and increases the amount of chromophores groups [163,164]. As an alternative, the steam and heat treatment of wood (steam explosion procedure) for the extraction of lignin, consists in heating the starting material at temperatures between 180 °C and 220 °C and high pressure. This procedure affords an effective discoloration of lignin [165]. In this latter case, the degree of color change (as evaluated by the DE* value) was highly dependent from the temperature and pressure parameters.

### 3.4. Chemical and UV Whitening of Lignin

Traditional chemical bleaching procedures are not suitable for lignin color reduction as their aim is to profoundly degrade the lignin structure. Softener procedures are therefore required to whiten lignin without reducing the antioxidant and UV shielding capability. Among them, sulfonation, sulphomethylation, and butane sulfonation treatments at high temperature and pressure have been shown to be effective in whitening eucalyptus lignin [166]. The reaction occurred preferentially at the C-α position of the phenylpropanoid side chain, while, when performed in the presence of formaldehyde and Na_2_SO_3_, the aromatic C-5 position was also modified [167]. UV irradiation combined with a long-time (20 h) H_2_O_2_ treatment effectively decolorizes sulfonated alkali lignin (SAL) by reducing the amounts of aromatic moieties and methoxyl and phenolic hydroxyl groups. Thanks to this treatment, SAL was successfully used as a dye dispersant [168]. Pre-treatments of the starting material can facilitate the bleaching procedure. They include autohydrolysis, steam explosion, and treatment with dilute acids. In particular, the autohydrolysis process improved the whitening of KL and decreased the excessive consumption of the sample during the overall whitening processes [169]. Selective functionalization procedures can also be applied in the whitening of lignin. For example, the acetylation of solvent-fractionated KL with acetic anhydride reduced the color intensity of the sample without interfering with its UV absorption capability (313.5% and 145.6% in brightness and L* value, respectively). In this latter case, the acetylation process was responsible for the inhibition of the auxochrome effect of the phenolic moieties, as a consequence of its electron withdrawing effect. Acetylation prevented demulsification processes due to the fine control of the polarity of lignin [158]. As a general trend the lightening process slightly interferes with the antioxidant and UV shielding properties. 

## 4. Conclusions

The future trend in the application of lignin in solar screen formulations appears to be dependent on two main variables: (a) the availability of adequate technologies for lignin transformation and enhancement; and (b) the availability of an economically sustainable and stable market over time. In the first case, different nanotechnologies are presently available for the transformation of technical lignin into nanoparticles with a controlled dimension and superficial charge, which are useful for the formulation of solar screens. The undoubted advantage of these new active ingredients lies in their proven biocompatibility, complete biodegradability, and antimicrobial activity, associated with improved antioxidant and UV shielding properties, emerging from the reduction of the molecular scale as a consequence of specific no covalent interactions. The great variety of lignin wastes from pulp and paper, agro-industrial, and biorefinery transformations also allows for a large panel of starting materials that differ in the solubility and other chemical and physical properties favoring compatibility with the ingredients of the formulation. In addition, lignin has peculiar UV absorbing properties which cover both the UV-A and UV-B range, thus reducing the actual requirements of complex mixtures of synthetic organic and inorganic filters. Being empty, the beneficial properties of lignin nanoparticle may be further increased by drug delivery processes involving the time-dependent release of selected natural substances [170]. Finally, a careful choice of the starting material, combined with enzymatic or chemical pre-treatments, also allows the control of the color of the nanoparticles, better meeting the consumer’s tastes. With regard to the sustainability of the economic market, the lignin industry demand is expected to register around 6% of compound annual growth rate (CAGR) between 2020 and 2026, and as a case of study, the demand for lignin-sulphonate wastes from pulp and paper is expected to grow at a CAGR of 1.6% over the forecast period, mainly for application in paint and coating products, laundry and cleaning detergents, biomaterials, and cosmetic and cosmeceutical formulations [171]. Processes for the extraction and recovery of technical lignin are available at the industrial scale as a result of collaboration between universities and private groups [172], and high quality grade lignin is available at approximately 600 $/t, a cost that is significantly lower than that of current sunscreens, also considering the benefit for human health and the environment [55]. Taken together, these data highlight lignin as one of the main alternatives for petroleum-based compounds in cosmeceutical and sunscreen formulations.

## Figures and Tables

**Table 1:** Toxic effects of some of the most representative OCFs applied in sunscreen formulations.

OCFs	UV Range	Photodegradation	Toxic Effects
AVOB	357	yes	Suppression of human trophoblast cells and apoptosis mediated by mitochondrial disruption [27], induction of obesogenic phenotypes [28], and hormone-like activity [29].
HS	295–315	no	Skin penetration [30], disrupts estrogen [31].
OMC	280–355	yes	Skin penetration, hormone like-activity; reproductive system, thyroid, and behavioral alterations in animal studies [17].
OB	270–350	yes	Estrogenic activity, alteration of sperm production in the animal associated with endometriosis in women [32].

AVOB: avobenzone. HS: homosalate. OMC: octyl methoxycinnamate. OB: oxybenzone.

**Table 2:** Cosmetic and cosmeceutical application of lignin.

Lignin Property	Petroleum Derived Compounds Substituted by Lignin	Ref.
Antioxidant	BHT, BHA	[66]
UV booster	acrylates/c10-30 alkyl acrylate cross-polymer	[67]
Antimicrobic agent	phenoxyethanol, hydroxybenzoates and triclosan	[68]
Chelating agent	EDTA, THPE	[69]
Emulsifier and stabilizer	acrylamides salts	[70]

BHT: butylated hydroxytoluene; BHA: butylated hydroxyanisole, EDTA: ethylenediaminetetraacetic acid; THPE: tetrahydroxypropyl ethylenediamine.

**Table 3:** Classification of different types of lignin depending on the amount of para-hydroxyphenyl (H), guaiacyl (G), and syringyl (S) sub-units.

Type	Grass Lignin	Softwood	Hardwood
H	5–35%	<5%	0–8%
G	35–80%	>95%	25–50%
S	20–55%	0%	45–75%

Proportion of different monolignols in lignin of different plants source. Adapted from [78,79,80].

**Table 4:** Major technologies available for the production of LNPs.

Lignin	Tech.	Size (nm)	pH	Shape	Advantages	Application(s)	Limits	Ref.
SL	SEP	50–250	7	S	no aggregation	drug delivery and stabilizer	toxic chemicals	[109,110]
SL	AP	50–250	>7	I	-	drug delivery, bioplastic	-	[111,112]
AL	AFR	30–100	<12	S	high yield	bioplastic	aggregate	[94,113,114]
AL	AP	30–100	<12	S	high stability	sunscreen	high ionic strength	[115]
AL	MR	30–100	<12	S	dispersibility	antioxidant, antimicrobial	toxic chemicals	[116,117]
AL	SEP	30–100	<12	S	-	antioxidant, antimicrobial	toxic chemicals	[115,118]
KL	AFR	38–250	4–12	I	stability	adhesives	-	[113,119]
KL	PCA	38–250	4–12	S	high solubility	commodities	high ionic strength	[120,121]
KL	FP	38–250	4–12	S	UV-shielding	materials	-	[110,122]
KL	MP	38–250	4–12	S	-	commodities	-	[110,122]
OL	AFR	30–250	3.5–8	S	high yield	adhesives	aggregate	[110,113,116]
OL	FP	30–250	3.5–8	S	stability	commodities	high ionic strength	[122]
OL	SEP	30–250	3.5–8	S	stability	materials	toxic chemicals	[112]

S: spherical shape. I: irregular shape. SEP: solvent exchange precipitation. AP: acid precipitation. AFR: aerosol flow reactor. MR: microchannel reactor. PCA: compressed fluid antisolvent technique. FP: flash-precipitation. MP: mini-emulsion polymerization.

**Table 5:** Antioxidant activity comparison of main lignin subunit.

#	Name	Structure	ARP ^a^	NRD ^b^	#	Name	Structure	ARP	NRD
I	Guaiacol	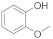	2.6	1.3	VI	Guaiacyl propanol-1	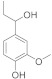	3.0	1.5
II	Syringol	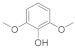	3.6	1.8	VII	Coniferyl alcohol	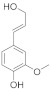	4.2	2.1
III	Guacyl propanone-1	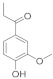	0.2	<0.1	VIII	Coniferyl aldehyde	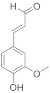	1.9	0.97
IV	propiosyringone	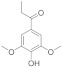	0.5	0.2	IX	Isoeugenol	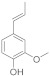	2.2	1
V	Propyl guaiacol	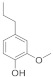	3.5	1.75	X	Eugenol	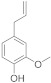	4	2

^a^ ARP: antiradical power, reverse of the dose inhibiting the 50% of the DPPH radical. ^b^ NRD: number of reduced DPPH units, moles of DPPH reduced per mole of compound.

## References

[1] Lehmann P. (2011). Sun exposed skin disease. Clin. Dermatol..

[2] Sambandan D.R., Ratner D. (2011). Sunscreens: An overview and update. J. Am. Acad. Dermatol..

[3] Bessada S.M.F., Alves R.C., Oliveira M.B.P.P. (2018). Coffee silverskin: A review on potential cosmetic applications. Cosmetics.

[4] Kawczyk-Krupka A., Bugaj A.M., Latos W., Zaremba K., Sieroń A. (2013). Photodynamic therapy in treatment of cutaneous and choroidal melanoma. Photodiagn. Photodyn. Ther..

[5] Abdul-Karim R.M., Cowey C.L. (2017). Challenging the standard of care in advanced melanoma: Focus on pembrolizumab. Cancer Manag. Res..

[6] Rodil R., Moeder M., Altenburger R., Schmitt-Jansen M. (2009). Photostability and phytotoxicity of selected sunscreen agents and their degradation mixtures in water. Anal. Bioanal. Chem..

[7] Rastogi S.C. (2002). UV filters in sunscreen products—A survey. Contact Dermat..

[8] Battistin M., Dissette V., Bonetto A., Durini E., Manfredini S., Marcomini A., Casagrande E., Brunetta A., Ziosi P., Molesini S. (2020). A new approach to UV protection by direct surface functionalization of TiO_2_ with the antioxidant polyphenol dihydroxyphenyl benzimidazole carboxylic acid. Nanomaterials.

[9] Sohn M. (2016). UV booster and photoprotection. Principles and Practice of Photoprotection.

[10] Juliano C., Magrini G.A. (2017). Cosmetic ingredients as emerging pollutants of environmental and health concern. A mini-review. Cosmetics.

[11] Senathirajah K., Attwood S., Bhagwat G., Carbery M., Wilson S., Palanisami T. (2021). Estimation of the mass of microplastics ingested—A pivotal first step towards human health risk assessment. J. Hazard. Mater..

[12] Sánchez-Quiles D., Tovar-Sánchez A. (2015). Are sunscreens a new environmental risk associated with coastal tourism?. Environ. Int..

[13] Gago-Ferrero P., Díaz-Cruz M.S., Barceló D. (2012). An overview of UV-absorbing compounds (organic UV filters) in aquatic biota. Anal. Bioanal. Chem..

[14] Corinaldesi C., Marcellini F., Nepote E., Damiani E., Danovaro R. (2018). Impact of inorganic UV filters contained in sunscreen products on tropical stony corals (*Acropora* spp.). Sci. Total Environ..

[15] Menard A., Drobne D., Jemec A. (2011). Ecotoxicity of nanosized TiO_2_. Review of in vivo data. Environ. Pollut..

[16] Huang Y., Law J.C.F., Lam T.K., Leung K.S.Y. (2021). Risks of organic UV filters: A review of environmental and human health concern studies. Sci. Total Environ..

[17] Nash J.F., Tanner P.R. (2014). Relevance of UV filter/sunscreen product photostability to human safety. Photodermatol. Photoimmunol. Photomed..

[18] Santos A.J.M., Miranda M.S., Esteves da Silva J.C.G. (2012). The degradation products of UV filters in aqueous and chlorinated aqueous solutions. Water Res..

[19] Damiani E., Astolfi P., Giesinger J., Ehlis T., Herzog B., Greci L., Baschong W. (2010). Assessment of the photo-degradation of UV-filters and radical-induced peroxidation in cosmetic sunscreen formulations. Free Radic. Res..

[20] Lionetti N., Rigano L. (2017). The New Sunscreens among Formulation Strategy, Stability Issues, Changing Norms, Safety and Efficacy Evaluations. Cosmetics.

[21] Stein H.V., Berg C.J., Maung J.N., O’Connor L.E., Pagano A.E., Macmanus-Spencer L.A., Paulick M.G. (2017). Photolysis and cellular toxicities of the organic ultraviolet filter chemical octyl methoxycinnamate and its photoproducts. Environ. Sci. Process. Impacts.

[22] Lorigo M., Cairrao E. (2019). Antioxidants as stabilizers of UV filters: An example for the UV-B filter octylmethoxycinnamate. Biomed. Dermatology.

[23] Hanson K.M., Gratton E., Bardeen C.J. (2006). Sunscreen enhancement of UV-induced reactive oxygen species in the skin. Free Radic. Biol. Med..

[24] Napper I.E., Bakir A., Rowland S.J., Thompson R.C. (2015). Characterisation, quantity and sorptive properties of microplastics extracted from cosmetics. Mar. Pollut. Bull..

[25] Ward J.B., Casagrande S.S., Cowie C.C. (2020). Urinary phenols and parabens and diabetes among US adults, NHANES 2005–2014. Nutr. Metab. Cardiovasc. Dis..

[26] Kapelewska J., Kotowska U., Wiśniewska K. (2016). Determination of personal care products and hormones in leachate and groundwater from Polish MSW landfills by ultrasound-assisted emulsification microextraction and GC-MS. Environ. Sci. Pollut. Res..

[27] Yang C., Lim W., Bazer F.W., Song G. (2018). Avobenzone suppresses proliferative activity of human trophoblast cells and induces apoptosis mediated by mitochondrial disruption. Reprod. Toxicol..

[28] Ahn S., An S., Lee M., Lee E., Pyo J.J., Kim J.H., Ki M.W., Jin S.H., Ha J., Noh M. (2019). A long-wave UVA filter avobenzone induces obesogenic phenotypes in normal human epidermal keratinocytes and mesenchymal stem cells. Arch. Toxicol..

[29] Klopčič I., Dolenc M.S. (2017). Endocrine activity of AVB, 2MR, BHA, and their mixtures. Toxicol. Sci..

[30] Ruszkiewicz J.A., Pinkas A., Ferrer B., Peres T.V., Tsatsakis A., Aschner M. (2017). Neurotoxic effect of active ingredients in sunscreen products, a contemporary review. Toxicol. Rep..

[31] Janjua N.R., Mogensen B., Andersson A.M., Petersen J.H., Henriksen M., Skakkebæk N.E., Wulf H.C. (2004). Systemic absorption of the sunscreens benzophenone-3, octyl- methoxycinnamate, and 3-(4-methyl-benzylidene) camphor after whole-body topical application and reproductive hormone levels in humans. J. Investig. Dermatol..

[32] Kunisue T., Chen Z., Buck Louis G.M., Sundaram R., Hediger M.L., Sun L., Kannan K. (2012). Urinary concentrations of benzophenone-type UV filters in U.S. women and their association with endometriosis. Environ. Sci. Technol..

[33] Stiefel C., Schwack W. (2014). Photoprotection in changing times—UV filter efficacy and safety, sensitization processes and regulatory aspects. Cosmet. Sci..

[34] Egambaram O.P., Kesavan Pillai S., Ray S.S. (2020). Materials Science Challenges in Skin UV Protection: A Review. Photochem. Photobiol..

[35] Rojas J., Londoño C., Ciro Y. (2016). The health benefits of natural skin uva photoprotective compounds found in botanical sources. Int. J. Pharm. Pharm. Sci..

[36] Morocho-Jácome A.L., Freire T.B., de Oliveira A.C., de Almeida T.S., Rosado C., Velasco M.V.R., Baby A.R. In Vivo SPF from multifunctional sunscreen systems developed with natural compounds—A review. J. Cosmet. Dermatol..

[37] Korkina L., Kostyuk V., Potapovich A., Mayer W., Talib N., De Luca C. (2018). Secondary plant metabolites for sun protective cosmetics: From pre-selection to product formulation. Cosmetics.

[38] Korkina L.G., Mayer W., de Luca C. (2017). Meristem plant cells as a sustainable source of redox actives for skin rejuvenation. Biomolecules.

[39] Takshak S., Agrawal S.B. (2019). Defense potential of secondary metabolites in medicinal plants under UV-B stress. J. Photochem. Photobiol. B Biol..

[40] Cavinato M., Waltenberger B., Baraldo G., Grade C.V.C., Stuppner H., Jansen-Dürr P. (2017). Plant extracts and natural compounds used against UVB-induced photoaging. Biogerontology.

[41] Nishida Y., Kumagai Y., Michiba S., Yasui H., Kishimura H. (2020). Efficient extraction and antioxidant capacity of mycosporine-like amino acids from red alga dulse palmaria palmata in Japan. Mar. Drugs.

[42] Whittock A.L., Turner M.A.P., Coxon D.J.L., Woolley J.M., Horbury M.D., Stavros V.G. (2020). Reinvestigating the Photoprotection Properties of a Mycosporine Amino Acid Motif. Front. Chem..

[43] Chrapusta E., Kaminski A., Duchnik K., Bober B., Adamski M., Bialczyk J. (2017). Mycosporine-Like Amino Acids: Potential health and beauty ingredients. Mar. Drugs.

[44] Pangestuti R., Siahaan E.A., Kim S.K. (2018). Photoprotective substances derived from marine algae. Mar. Drugs.

[45] Chiari B.G., Trovatti E., Pecoraro É., Corrêa M.A., Cicarelli R.M.B., Ribeiro S.J.L., Isaac V.L.B. (2014). Synergistic effect of green coffee oil and synthetic sunscreen for health care application. Ind. Crops Prod..

[46] Tomaino A., Cristani M., Cimino F., Speciale A., Trombetta D., Bonina F., Saija A. (2006). In vitro protective effect of a Jacquez grapes wine extract on UVB-induced skin damage. Toxicol. In Vitro.

[47] Huynh A., Abou-Dahech M.S., Reddy C.M., O’Neil G.W., Chandler M., Baki G. (2019). Alkenones, a renewably sourced, biobased wax as an SPF booster for organic sunscreens. Cosmetics.

[48] Ragauskas A.J., Beckham G.T., Biddy M.J., Chandra R., Chen F., Davis M.F., Davison B.H., Dixon R.A., Gilna P., Keller M. (2014). Lignin valorization: Improving lignin processing in the biorefinery. Science.

[49] Calcio Gaudino E., Tabasso S., Grillo G., Cravotto G., Dreyer T., Schories G., Altenberg S., Lauberte L., Telysheva G. (2018). Wheat straw lignin extraction with bio-based solvents using enabling technologies. Comptes Rendus Chem..

[50] Xie J., Hse C.-Y., Shupe T.F., Hu T. (2015). Physicochemical characterization of lignin recovered from microwave-assisted delignified lignocellulosic biomass for use in biobased materials. J. Appl. Polym. Sci..

[51] Brahim M., Checa Fernandez B.L., Regnier O., Boussetta N., Grimi N., Sarazin C., Husson C., Vorobiev E., Brosse N. (2017). Impact of ultrasounds and high voltage electrical discharges on physico-chemical properties of rapeseed straw’s lignin and pulps. Bioresour. Technol..

[52] Gordobil O., Herrera R., Yahyaoui M., Ilk S., Kaya M., Labidi J. (2018). Potential use of kraft and organosolv lignins as a natural additive for healthcare products. RSC Adv..

[53] Norgren M., Edlund H. (2014). Lignin: Recent advances and emerging applications. Curr. Opin. Coll. Interface Sci..

[54] Stewart D. (2008). Lignin as a base material for materials applications: Chemistry, application and economics. Ind. Crops Prod..

[55] Vishtal A., Kraslawski A. (2011). Challenges in industrial applications of technical lignins. BioResources.

[56] Lendlein A., Balk M., Tarazona N.A., Oliver E.C. (2019). Gould Bioperspectives for Shape-Memory Polymers as Shape Programmable. Act. Mater..

[57] Yuan T.Q., Xu F., Sun R.C. (2013). Role of lignin in a biorefinery: Separation characterization and valorization. J. Chem. Technol. Biotechnol..

[58] Azadi P., Inderwildi O.R., Farnood R., King D.A. (2013). Liquid fuels, hydrogen and chemicals from lignin: A critical review. Renew. Sustain. Energy Rev..

[59] Liao J.J., Latif N.H.A., Trache D., Brosse N., Hussin M.H. (2020). Current advancement on the isolation, characterization and application of lignin. Int. J. Biol. Macromol..

[60] Peart C., Ni Y. (2001). UV-Vis spectra of lignin model compounds in the presence of metal ions and chelants. J. Wood Chem. Technol..

[61] Mahmood Z., Yameen M., Jahangeer M., Riaz M., Ghaffar A., Javid I. (2018). Lignin as Natural Antioxidant Capacity. Lignin—Trends and Applications.

[62] García A., González Alriols M., Spigno G., Labidi J. (2012). Lignin as natural radical scavenger. Effect of the obtaining and purification processes on the antioxidant behaviour of lignin. Biochem. Eng. J..

[63] Yu O., Ho Kim K. (2020). Lignin to Materials: A Focused Review on Recent Novel Lignin Applications. Appl. Sci..

[64] (2021). In Vivo Toxicity Assessment of Chitosan-Coated Lignin Nanoparticles in Embryonic Zebrafish (*Danio rerio*). Nanomaterials.

[65] Wang B., Sun D., Wang H.M., Yuan T.Q., Sun R.C. (2019). Green and Facile Preparation of Regular Lignin Nanoparticles with High Yield and Their Natural Broad-Spectrum Sunscreens. ACS Sustain. Chem. Eng..

[66] Ratanasumarn N., Chitprasert P. (2020). Cosmetic potential of lignin extracts from alkaline-treated sugarcane bagasse: Optimization of extraction conditions using re-sponse surface methodology. Int. J. Biol. Macromol..

[67] Gordobil O., Olaizola P., Banales M.J., Labidi J. (2020). Lignins from Agroindustrial by-Products as Natural Ingredients for Cosmetics: Chemical Structure and In Vitro Sunscreen and Cytotoxic Activities. Molecules.

[68] Espinoza-Acosta J.L., Torres-Chávez P.I., Ramírez-Wong B., López-Saiz C.M., Montaño-Leyva B. (2016). Antioxidant, antimicrobial, and antimutagenic properties of technical lignins and their applications. BioResources.

[69] Jardim J.M., Hart P.W., Lucia L., Jameel H. (2020). Insights into the Potential of Hardwood Kraft Lignin to Be a Green Platform Material for Emergence of the Biorefinery. Polymers.

[70] Kirsi S. (2020). Mikkonen. Strategies for structuring diverse emulsion systems by using wood lignocellulose-derived stabilizers. Green Chem..

[71] Kleinert M., Barth T. (2008). Phenols from lignin. Chem. Eng. Technol..

[72] Martínez Á.T., Rencoret J., Marques G., Gutiérrez A., Ibarra D., Jiménez-Barbero J., del Río J.C. (2008). Monolignol acylation and lignin structure in some nonwoody plants: A 2D NMR study. Phytochemistry.

[73] Guerriero G., Hausman J.F., Strauss J., Ertan H., Siddiqui K.S. (2016). Lignocellulosic biomass: Biosynthesis, degradation, and industrial utilization. Eng. Life Sci..

[74] Wang Y., Chantreau M., Sibout R., Hawkins S. (2013). Plant cell wall lignification and monolignol metabolism. Front. Plant Sci..

[75] Davin L.B., Lewis N.G. (2005). Lignin primary structures and dirigent sites. Curr. Opin. Biotechnol..

[76] Crestini C., Melone F., Sette M., Saladino R. (2011). Milled wood lignin: A linear oligomer. Biomacromolecules.

[77] Liu Q., Luo L., Zheng L. (2018). Lignins: Biosynthesis and biological functions in plants. Int. J. Mol. Sci..

[78] Gellerstedt G., Henriksson G. (2008). Monomers, Polymers and Composites from Renewable Resources.

[79] Schutyser W., Renders T., Van den Bossche G., Van den Bosch S., Koelewijn S.-F., Ennaert T., Sels B.F., Van de Voorde M., Sels B. (2017). Catalysis in Lignocellulosic Biorefineries: The Case of Lignin Conversion. Nanotechnology in Catalysis.

[80] Wang H., Male J., Wang Y. (2013). Recent advances in hydrotreating of pyrolysis bio-oil and its oxygen-containing model compounds. ACS Catal..

[81] Giummarella N., Pu Y., Ragauskas A.J., Lawoko M. (2019). A critical review on the analysis of lignin carbohydrate bonds. Green Chem..

[82] Duval A., Lawoko M., Hatakka P.A., Maciejewska A., Veringa H., Sanders J., Peteves S.D., Fernandes E.M., Pires R.A., Mano J.F. (2014). A review on lignin-based polymeric, micro- and nano-structured materials. React. Funct. Polym..

[83] Espinoza-Acosta J.L., Torres-Chávez P.I., Carvajal-Millán E., Ramírez-Wong B., Bello-Pérez L.A., Montaño-Leyva B. (2014). Ionic liquids and organic solvents for recovering lignin from lignocellulosic biomass. BioResources.

[84] Baurhoo B., Phillip L., Ruiz-Feria C.A. (2007). Effects of purified lignin and mannan oligosaccharides on intestinal integrity and microbial populations in the ceca and litter of broiler chickens. Poult. Sci..

[85] Baurhoo B., Ruiz-Feria C.A., Zhao X. (2008). Purified lignin: Nutritional and health impacts on farm animals-A review. Anim. Feed Sci. Technol..

[86] Calvo-Flores F.G., Dobado J.A. (2010). Lignin as renewable raw material. ChemSusChem.

[87] Eastwood M., Kritchevsky D. (2005). Dietary fiber: How did we get where we are?. Annu. Rev. Nutr..

[88] Nasrullah A., Bhat A.H., Isa M.H. (2016). Lignin: A sustainable biosorbent for heavy metal adsorption from wastewater, a review. AIP Conf. Proc..

[89] Vinardell M.P., Ugartondo V., Mitjans M. (2008). Potential applications of antioxidant lignins from different sources. Ind. Crops Prod..

[90] Yamamoto Y., Shirono H., Kono K., Ohashi Y. (1997). Immunopotentiating activity of the water-soluble lignin rich fraction prepared from lem—The extract of the solid culture medium of lentinus edodes mycelia. Biosci. Biotechnol. Biochem..

[91] Vinardell M.P., Mitjans M. (2017). Lignins and their derivatives with beneficial effects on human health. Int. J. Mol. Sci..

[92] Evstigneyev E.I., Shevchenko S.M. (2019). Structure, chemical reactivity and solubility of lignin: A fresh look. Wood Sci. Technol..

[93] Mishra P.K., Ekielski A. (2019). The Self-Assembly of Lignin and Its Application in Nanoparticle Synthesis: A Short Review. Nanomaterials.

[94] Yearla S.R., Padmasree K. (2016). Preparation and characterisation of lignin nanoparticles: Evaluation of their potential as antioxidants and UV protectants. J. Exp. Nanosci..

[95] Alqahtani M.S., Alqahtani A., Al-Thabit A., Roni M., Syed R. (2019). Novel lignin nanoparticles for oral drug delivery. J. Mater. Chem. B.

[96] Chollet B., Lopez-Cuesta J.M., Laoutid F., Ferry L. (2019). Lignin nanoparticles as a promising way for enhancing lignin flame retardant effect in polylactide. Materials.

[97] Yang W., Rallini M., Wang D.Y., Gao D., Dominici F., Torre L., Kenny J.M., Puglia D. (2018). Role of lignin nanoparticles in UV resistance, thermal and mechanical performance of PMMA nanocomposites prepared by a combined free-radical graft polymerization/masterbatch procedure. Compos. Part A Appl. Sci. Manuf..

[98] Rivière G.N., Korpi A., Sipponen M.H., Zou T., Kostiainen M.A., Österberg M. (2020). Agglomeration of Viruses by Cationic Lignin Particles for Facilitated Water Purification. ACS Sustain. Chem. Eng..

[99] Zhang X., Yang M., Yuan Q., Cheng G. (2019). Controlled Preparation of Corncob Lignin Nanoparticles and their Size-Dependent Antioxidant Properties: Toward High Value Utilization of Lignin. ACS Sustain. Chem. Eng..

[100] Garver T.M., Callaghan P.T. (1991). Hydrodynamics of Kraft Lignins. Macromolecules.

[101] Lu Y., Fan H., Stump A., Ward T.L., Rieker T., Brinker C.J. (1999). Aerosol-assisted self-assembly of mesostructured spherical nanoparticles. Nature.

[102] Borisova O.V., Billon L., Cernochova Z., Lapp A., Stepanek P., Borisov O.V. (2015). Effect of temperature on self-assembly of amphiphilic block-gradient copolymers of styrene and acrylic acid. Macromol. Symp..

[103] Guerra A., Gaspar A.R., Contreras S., Lucia L.A., Crestini C., Argyropoulos D.S. (2007). On the propensity of lignin to associate: A size exclusion chromatography study with lignin derivatives isolated from different plant species. Phytochemistry.

[104] Deng Y., Feng X., Yang D., Yi C., Qiu X. (2012). π-π Stacking of the aromatic groups in lignosulfonates. BioResources.

[105] Zhao W., Xiao L.P., Song G., Sun R.C., He L., Singh S., Simmons B.A., Cheng G. (2017). From lignin subunits to aggregates: Insights into lignin solubilization. Green Chem..

[106] Whitesides G.M., Boncheva M. (2002). Beyond molecules: Self-assembly of mesoscopic and macroscopic components. Proc. Natl. Acad. Sci. USA.

[107] Lindström T. (1980). The colloidal behaviour of kraft lignin. Colloid Polym. Sci..

[108] Lindström T., Westman L. (1982). The colloidal behaviour of kraft lignin. Colloid Polym. Sci..

[109] Gupta A.K., Mohanty S., Nayak S.K. (2015). Synthesis, Characterization and Application of Lignin Nanoparticles (LNPs). Mater. Focus.

[110] Low L.E., Teh K.C., Siva S.P., Chew I.M.L., Mwangi W.W., Chew C.L., Goh B.H., Chan E.S., Tey B.T. (2021). Lignin nanoparticles: The next green nanoreinforcer with wide opportunity. Environ. Nanotechnol. Monit. Manag..

[111] Frangville C., Rutkevičius M., Richter A.P., Velev O.D., Stoyanov S.D., Paunov V.N. (2012). Fabrication of environmentally biodegradable lignin nanoparticles. ChemPhysChem.

[112] Chowdhury M.A. (2014). The controlled release of bioactive compounds from lignin and lignin-based biopolymer matrices. Int. J. Biol. Macromol..

[113] Ago M., Huan S., Borghei M., Raula J., Kauppinen E.I., Rojas O.J. (2016). High-throughput synthesis of lignin particles (~30 nm to ~2 μm) via aerosol flow reactor: Size fractionation and utilization in pickering emulsions. ACS Appl. Mater. Interfaces.

[114] Chen L., Zhou X., Shi Y., Gao B., Wu J., Kirk T.B., Xu J., Xue W. (2018). Green synthesis of lignin nanoparticle in aqueous hydrotropic solution toward broadening the window for its processing and application. Chem. Eng. J..

[115] Yang W., Fortunati E., Gao D., Balestra G.M., Giovanale G., He X., Torre L., Kenny J.M., Puglia D. (2018). Valorization of Acid Isolated High Yield Lignin Nanoparticles as Innovative Antioxidant/Antimicrobial Organic Materials. ACS Sustain. Chem. Eng..

[116] Ju T., Zhang Z., Li Y., Miao X., Ji J. (2019). Continuous production of lignin nanoparticles using a microchannel reactor and its application in UV-shielding films. RSC Adv..

[117] Beisl S., Friedl A., Miltner A. (2017). Lignin from micro- to nanosize: Applications. Int. J. Mol. Sci..

[118] Yang J., Ching Y.C., Chuah C.H. (2019). Applications of lignocellulosic fibers and lignin in bioplastics: A review. Polymers.

[119] Myint A.A., Lee H.W., Seo B., Son W.S., Yoon J., Yoon T.J., Park H.J., Yu J., Yoon J., Lee Y.W. (2016). One pot synthesis of environmentally friendly lignin nanoparticles with compressed liquid carbon dioxide as an antisolvent. Green Chem..

[120] Yiamsawas D., Beckers S.J., Lu H., Landfester K., Wurm F.R. (2017). Morphology-Controlled Synthesis of Lignin Nanocarriers for Drug Delivery and Carbon Materials. ACS Biomater. Sci. Eng..

[121] Richter A.P., Bharti B., Armstrong H.B., Brown J.S., Plemmons D., Paunov V.N., Stoyanov S.D., Velev O.D. (2016). Synthesis and characterization of biodegradable lignin nanoparticles with tunable surface properties. Langmuir.

[122] Beisl S., Miltner A., Friedl A. (2017). Lignin from micro- to nanosize: Production methods. Int. J. Mol. Sci..

[123] Sauce R., Aparecida Sales de Oliveira Pinto C., Robles Velasco M.V., Rosado C., Rolim Baby A. (2021). Ex Vivo penetration analysis and anti-inflammatory efficacy of the association of ferulic acid and UV filters. Eur. J. Pharm. Sci..

[124] De Lima Cherubim D.J., Buzanello Martins C.V., Oliveira Fariña L., da Silva de Lucca R.A. (2020). Polyphenols as natural antioxidants in cosmetics applications. J. Cosmet. Dermatol..

[125] Dizhbite T., Telysheva G., Jurkjane V., Viesturs U. (2004). Characterization of the radical scavenging activity of lignins—Natural antioxidants. Bioresour. Technol..

[126] Ibrahim M.N.M., Iqbal A., Shen C.C., Bhawani S.A., Adam F. (2019). Synthesis of lignin based composites of TiO_2_ for potential application as radical scavengers in sunscreen formulation. BMC Chem..

[127] Freitas F.M.C., Cerqueira M.A., Gonçalves C., Azinheiro S., Garrido-Maestu A., Vicente A.A., Pastrana L.M., Teixeira J.A., Michelin M. (2020). Green synthesis of lignin nano- and micro-particles: Physicochemical characterization, bioactive properties and cytotoxicity assessment. Int. J. Biol. Macromol..

[128] Pylypchuk I.V., Lindén P.A., Lindström M.E., Sevastyanova O. (2020). New Insight into the Surface Structure of Lignin Nanoparticles Revealed by 1H Liquid-State NMR Spectroscopy. ACS Sustain. Chem. Eng..

[129] Piccinino D., Capecchi E., Botta L., Bizzarri B.M., Bollella P., Antiochia R., Saladino R. (2018). Layer-by-Layer Preparation of Microcapsules and Nanocapsules of Mixed Polyphenols with High Antioxidant and UV-Shielding Properties. Biomacromolecules.

[130] Trevisan H., Rezende C.A. (2020). Pure, stable and highly antioxidant lignin nanoparticles from elephant grass. Ind. Crops Prod..

[131] Qian Y., Zhong X., Li Y., Qiu X. (2017). Fabrication of uniform lignin colloidal spheres for developing natural broad-spectrum sunscreens with high sun protection factor. Ind. Crops Prod..

[132] Morsella M., D’Alessandro N., Lanterna A.E., Scaiano J.C. (2016). Improving the Sunscreen Properties of TiO_2_ through an Understanding of Its Catalytic Properties. ACS Omega.

[133] Widsten P., Tamminen T., Liitiä T. (2020). Natural Sunscreens Based on Nanoparticles of Modified Kraft Lignin (CatLignin). ACS Omega.

[134] Liu R., Dai L., Xu C., Wang K., Zheng C., Si C. (2020). Lignin-Based Micro- and Nanomaterials and their Composites in Biomedical Applications. ChemSusChem.

[135] Sadeghifar H., Ragauskas A. (2020). Lignin as a UV Light blocker-a review. Polymers.

[136] Tribulová T., Kacík F., Evtuguin D., Cabalová I. (2016). Assessment of chromophores in chemically treated and aged wood by uv-vis diffuse reflectance spectroscopy. Cellul. Chem. Technol..

[137] Wang M., Zhao Y., Li J. (2019). From hollow lignin microsphere preparation to simultaneous preparation of urea encapsulation for controlled release using industrial kraft lignin via slow and exhaustive acetone-water evaporation. Holzforschung.

[138] Ma Z., Liu C., Niu N., Chen Z., Li S., Liu S., Li J. (2018). Seeking Brightness from Nature: J-Aggregation-Induced Emission in Cellulolytic Enzyme Lignin Nanoparticles. ACS Sustain. Chem. Eng..

[139] Furman G.S., Lonsky W.F.W. (1988). Charge-transfer complexes in kraft lignin part 1: Occurrence. J. Wood Chem. Technol..

[140] Chang H.T., Su Y.C., Chang S.T. (2006). Studies on photostability of butyrylated, milled wood lignin using spectroscopic analyses. Polym. Degrad. Stab..

[141] Capecchi E., Piccinino D., Delfino I., Bollella P., Antiochia R., Saladino R. (2018). Functionalized tyrosinase-lignin nanoparticles as sustainable catalysts for the oxidation of phenols. Nanomaterials.

[142] Piccinino D., Capecchi E., Botta L., Bollella P., Antiochia R., Crucianelli M., Saladino R. (2019). Layer by layer supported laccase on lignin nanoparticles catalyzes the selective oxidation of alcohols to aldehydes. Catal. Sci. Technol..

[143] Li Y., Yang D., Lu S., Qiu X., Qian Y., Li P. (2019). Encapsulating TiO_2_ in Lignin-Based Colloidal Spheres for High Sunscreen Performance and Weak Photocatalytic Activity. ACS Sustain. Chem. Eng..

[144] Yaqoob Khan A., Talegaonkar S., Iqbal Z., Jalees Ahmed F., Krishan Khar R. (2006). Multiple Emulsions: An Overview. Curr. Drug Deliv..

[145] Benvegnu T., Plusquellec D., Lemiègre L., Belgacem M.N., Gandini A. (2008). Surfactants from renewable sources: Synthesis and applications. Monomers, Polymers and Composites from Renewable Resources.

[146] Ruwoldt J. (2020). A Critical Review of the Physicochemical Properties of Lignosulfonates: Chemical Structure and Behavior in Aqueous Solution, at Surfaces and Interfaces. Surfaces.

[147] Low L.E., Siva S.P., Ho Y.K., Chan E.S., Tey B.T. (2020). Recent advances of characterization techniques for the formation, physical properties and stability of Pickering emulsion. Adv. Colloid Interface Sci..

[148] Österberg M., Sipponen M.H., Mattos B.D., Rojas O.J. (2020). Spherical lignin particles: A review on their sustainability and applications. Green Chem..

[149] Oh-Hara T., Sakagami H., Kawazoe Y., Kaiya T., Komatsu N., Ohsawa N., Fujimaki M., Tanuma S.I., Konno K. (1990). Antimicrobial spectrum of lignin-related pine cone extracts of pinus parviflora Sieb. et Zucc. In Vivo.

[150] Sláviková E., Košíková B. (1994). Inhibitory effect of lignin by-products of pulping on yeast growth. Folia Microbiol. Off. J. Inst. Microbiol. Acad. Sci. Czech Repub..

[151] Richter A.P., Brown J.S., Bharti B., Wang A., Gangwal S., Houck K., Cohen Hubal E.A., Paunov V.N., Stoyanov S.D., Velev O.D. (2015). An environmentally benign antimicrobial nanoparticle based on a silver-infused lignin core. Nat. Nanotechnol..

[152] Mohan D., Pittman C.U., Steele P.H. (2006). Single, binary and multi-component adsorption of copper and cadmium from aqueous solutions on Kraft lignin—A biosorbent. J. Coll. Interface Sci..

[153] Wu Y., Zhang S., Guo X., Huang H. (2008). Adsorption of chromium (III) on lignin. Bioresour. Technol..

[154] Harmita H., Karthikeyan K.G., Pan X.J. (2009). Copper and cadmium sorption onto kraft and organosolv lignins. Bioresour. Technol..

[155] Guo X., Zhang S., Shan X.-Q. (2008). Adsorption of metal ions on lignin. J. Hazard. Mater..

[156] Oviedo C., Rodríguez J. (2003). EDTA: The chelating agent under environmental scrutiny. Quim. Nova.

[157] Wang J., Deng Y., Qian Y., Qiu X., Ren Y., Yang D. (2016). Reduction of lignin color via one-step UV irradiation. Green Chem..

[158] Li C., Ping Q., Shi H., Li N., Zhang J., Wang C. (2020). A rapid and quantitative method for assessing the whiteness of whitened lignin based on an in-depth analysis of reported methods. Int. J. Biol. Macromol..

[159] Vainio U., Maximova N., Hortling B., Laine J., Stenius P., Simola L.K., Gravitis J., Serimaa R. (2004). Morphology of dry lignins and size and shape of dissolved kraft lignin particles by X-ray scattering. Langmuir.

[160] Zhang H., Chen F., Liu X., Fu S. (2018). Micromorphology Influence on the Color Performance of Lignin and Its Application in Guiding the Preparation of Light-colored Lignin Sunscreen. ACS Sustain. Chem. Eng..

[161] Lee S.C., Yoo E., Lee S.H., Won K. (2020). Preparation and application of light-colored lignin nanoparticles for broad-spectrum sunscreens. Polymers.

[162] Hang H., Bai Y., Yu B., Liu X., Chen F. (2017). A practicable process for lignin color reduction: Fractionation of lignin using methanol/water as a solvent. Green Chem..

[163] Tjeerdsma B.F., Boonstra M., Pizzi A., Tekely P., Militz H. (1998). Characterisation of thermally modified wood: Molecular reasons for wood performance improvement. Holz Roh Werkst..

[164] Sundqvist B., Karlsson O., Westermark U. (2006). Determination of formic-acid and acetic acid concentrations formed during hydrothermal treatment of birch wood and its relation to colour, strength and hardness. Wood Sci. Technol..

[165] Zhang P., Wei Y., Liu Y., Gao J., Chen Y., Fan Y. (2018). Heat-induced discoloration of chromophore structures in Eucalyptus lignin. Materials.

[166] Zhang H., Bai Y., Zhou W., Chen F. (2017). Color reduction of sulfonated eucalyptus kraft lignin. Int. J. Biol. Macromol..

[167] Podschuna J., Saakea B., Lehnenb R. (2011). Reactivity enhancement of organosolv lignin by phenolation for improved bio-based thermosets. Eur. Polym. J..

[168] Matsushita Y., Yasuda S. (2005). Preparation and evaluation of lignosulfonates as a dispersant for gypsum paste from acid hydrolysis lignin. Bioresour. Technol..

[169] Zhang H., Liu X., Fu S., Chen Y. (2019). High-value utilization of kraft lignin: Color reduction and evaluation as sunscreen ingredient. Int. J. Biol. Macromol..

[170] Siddiqui L., Bag J., Seethab Mittal D., Leekha A., Mishra H., Mishra M., Verma A.K., Mishra P.K., Ekielski A., Iqbal Z. (2020). Assessing the potential of lignin nanoparticles as drug carrier: Synthesis, cytotoxicity and genotoxicity studies. Int. J. Biol. Macromol..

[171] Dessbesell L., Paleologou M., Leitch M., Xu C. (2020). Global lignin supply overview and kraft lignin potential as an alternative for petroleum-based polymers. Renew. Sustain. Energy Rev..

[172] Tomani P.E.R. (2010). The lignoboost process. Cellul. Chem. Technol..

